# HSF1 is a novel prognostic biomarker in high-risk prostate cancer that correlates with ferroptosis

**DOI:** 10.1007/s12672-023-00715-1

**Published:** 2023-06-23

**Authors:** GaoZhen Jia, WenBo Wu, Lei Chen, Yang Yu, QiLin Tang, HaiTao Liu, Qi Jiang, BangMin Han

**Affiliations:** Department of Urology, Shanghai General Hospital (Shanghai Peoples Hospital 1), Shanghai JiaoTong University School of Medicine, Shanghai, 200080 China

**Keywords:** High-risk prostate cancer, HSF1, Ferroptosis, HSPE1, RSL3

## Abstract

**Background:**

Prostate cancer (PC) is the most common cancer in older men in Europe and the United States and has the second highest death rate among male cancers. The transcription of heat shock proteins by Heat shock factor 1 (HSF1) is known to regulate cell growth and stress. Nevertheless, the impact of HSF1 on ferroptosis in PC through heat shock protein 10 (HSPE1) remains unexplored.

**Methods:**

This study employed a range of analytical techniques, including proteomics sequencing, LC–MS/MS, CHIP-qPCR, Western blotting, immunohisto -chemistry, JC-1, CKK-8, MDA, and ROS assays. Bioinformatics analysis was performed using the UALCAN,GEPIA, PCaDB and Metascape platforms.

**Results:**

Compared with levels observed in tumor-adjacent tissue, the levels of proteins associated with fatty acids, amino acids and the oxidative phosphorylation metabolic pathway were significantly upregulated in high-risk PC tissue (Gleason score ≥ 8). HSF1 mRNA and protein levels in high-risk PC tissues were significantly higher than those observed in medium-risk PC (Gleason score = 7) and low-risk PC (Gleason score ≤ 6) tissues. ssGSEA showed that HSF1 was involved in the proliferation and anti-apoptotic processes of PC. Further bioinformatics analysis showed that HSF1 potentially affects the mitochondrial oxidative phosphorylation (OXPHOS) system by targeting HSPE1. In addition, HSF1 alleviates ROS and MDA levels to enhance the resistance of prostate cancer cells to ferroptosis by regulating HSPE1 in vitro, and HSF1 knockout promotes the susceptibility of PC to RSL3 treatment by increasing ferroptosis in vivo.

**Conclusion:**

Collectively, our findings suggest that HSF1 exerts a significant influence on PC. HSF1 may represent a promising biomarker for identifying high-risk PC, and the elimination of HSF1 could potentially enhance the therapeutic effectiveness of RSL3.

## Introduction

Prostate cancer is prevalent among elderly men in Europe and the United States and has the second highest death rate among male cancers [1]. In the United States, approximately 14–24% of prostate cancer patients are at high risk at the time of diagnosis even after prostate-specific antigen (PSA) screening. High-risk prostate cancer is defined as a preoperative PSA level > 20 ng/mL, a Gleason score of ≥ 8, or clinical stage ≥ T2c [2, 3]. Prostate cancer patients with a high-risk profile exhibit significant heterogeneity, rendering the optimal treatment strategy uncertain. Despite the availability of several treatment options, including surgery and radiation therapy, the recurrence rate remains high regardless of treatment [4, 5]. Heat shock proteins (HSPs) are highly conserved in all mammalian cells and are crucial lines of defense for tumor cells against stress-related challenges. HSPs help tumors adapt to sudden changes in temperature and high levels of reactive oxygen species (ROS) and ensure accurate protein folding in tumor cells under stress conditions [6]. In this study, we characterized the expression of HSPs in high-risk PC for the first time. We found that HSF1 levels were significantly increased in high-risk PC and positively correlated with the Gleason score. Furthermore, the involvement of HSF1 in the ferroptosis of PC through the regulation of HSPE1 suggests its potential as a novel prognostic and therapeutic target for high-risk PC.

Heat shock factor 1 (HSF1) regulates cell growth, metabolism and stress responses by regulating the transcription of heat shock proteins. Common HSFs in the human body include HSF1, HSF2 and HSF4, among which HSF1 is the most important. HSF1 is significantly overexpressed in different tumors and associated with poor prognosis [7–9]. In 2000, Hoang, AT studied a pair of nonmetastatic and metastatic variant human prostate cell lines and found for the first time that HSF1 was significantly upregulated in malignant prostate epithelial cells and involved in cell growth, differentiation, and apoptosis [10]. Prostate cancer development and progression depend on androgen receptor (AR) signaling, and the HSF1-activated multichaperone complex (HSP90, HSP70, and HSP40) plays a crucial role in maintaining AR stability. In addition, studies have shown that HSF1 expression could serve as a novel prognostic marker for patient risk stratification of survival after radical prostatectomy and as a novel therapeutic target for castration-resistant prostate cancer (CRPC) [11–13]. The HSPE1 gene encodes the HSP10 protein, which assists in protein folding in the mitochondrial matrix, and the loss of HSPE1 significantly upregulates the mitochondrial superoxide levels, leading to ROS-related damage in cells [14]. In summary, the upregulation of HSF1 and HSPE1 may have significant implications in the development of prostate cancer, particularly in relation to cellular proliferation and defense against oxidative stress. Our investigation yielded novel findings, as we observed that the depletion of HSF1 in prostate cancer cell lines resulted in a decrease in HSPE1 expression and heightened susceptibility to ferroptosis triggered by the RSL3.

Ferroptosis is an iron-dependent form of nonapoptotic cell death. Previous studies showed that low celastrol and erastin concentrations activated HSF1 to protect cells from proteotoxic stress, and HSF1 knockdown further enhanced non-small cell lung cancer cell death in vitro and vivo. Another study in cardiomyocytes showed that HSF1 overexpression reduced palmitic acid (PA)-induced cell death and lipid peroxidation and maintained iron homeostasis by regulating iron metabolism-related genes [15, 16]. These findings indicate that HSF1 may serve as a crucial protective gene against ferroptosis. Our own research, which analyzed pathways in high-risk prostate cancer, demonstrated significant alterations in the expression of genes associated with oxidative phosphorylation and fatty acid metabolism, both of which are closely linked to mitochondrial function. HSPE1 participates in the assembly of intramitochondrial protein complexes and influences the production of mitochondrial reactive oxygen species. Consequently, HSF1/HSPE1 may serve as significant molecules in preserving energy metabolism and regulating the equilibrium between ROS generation and removal in high-risk PC.

In conclusion, our study has revealed a novel finding that HSF1 expression is notably upregulated in individuals with high-risk PC and exhibits a positive correlation with the Gleason score. Furthermore, our results indicate that HSF1 plays a crucial role in the ferroptosis of PC cells by modulating HSPE1, thereby highligh- ting its potential as a promising prognostic and therapeutic target for high-risk PC.

## Methods and materials

### Patient recruitment

All patient samples collected in this study were approved by the Shanghai General Hospital ethics committee, and 13 pairs of samples after radical prostatectomy and their corresponding paracancerous normal tissues were used for LC–MS/MS analysis (Gleason score ≥ 8). In addition, 30 clinical needle biopsy samples (normal = 3, Gleason score 6 = 10, Gleason score 7 = 14, Gleason score 8–9 = 15) were used for immunohistochemical analysis.

### LC–MS/MS and enrichment analysis

High-throughput proteomics LC–MS/MS assays were performed, and raw DIA data were processed using Spectronaut X (Biognosys AG, Switzerland) with default settings. After assessment by Student’s t test, differentially expressed proteins were filtered (Q value < 0.05, absolute AVG log2 ratio > 0.58). The Metascape platform was used for enrichment analysis [17].

### Detection of HSF1 protein expression by immunohistochemistry

Paraffin blocks were dewaxed and incubated with 3% H_2_O_2_ at room temperature for 5–10 min. The samples were then sealed with 5–10% normal goat serum (diluted with PBS) and incubated at room temperature for 10 min. The samples were sequentially incubated with a primary antibody targeting HSF1 (Abcam, ab52757) at 37 ℃ for 1 h followed by a working solution containing streptavidin labeled with horseradish or alkaline phosphatase at 37 ℃ for 10–30 min, and the chromogenic agent DAB was applied for 3–5 min.

### HSF1 mRNA expression and clinical prognosis

Correlations between HSF1 mRNA expression and clinical parameters of prostate cancer patients, including individual Gleason score and lymph node metastasis status, were analyzed by UALCAN (https://ualcan.path.uab.Edu), GEPIA (http://gepia.cancer-pku.cn/) and PCaDB (http://bioinfo.jialab-ucr.org/PCaDB/). Significant differences are indicated in the figures.

### Cell culture and HSF1 shRNA or HSPE1 plasmid transfection in vitro

PC3 and LNCaP cell lines were provided from the Cell Bank of the Chinese Academy of Sciences (Shanghai, China). Cultured with RPMI 1640 with 10% fetal bovine serum and 1% penicillin (GIBCO, Grand Island, NY, USA). These cells were cultured in a thermostat containing 5% CO_2_ at 37 °C. The cells were distributed into a six-well plate at a density of 5 × 10^5^ cells/ml per well. During the ferroptosis induction experiment, Erastin (S7242, Selleck, USA) and RSL3 (S8155,Selleck, USA) were dissolved in DMSO and subsequently introduced into the experimental medium at concentrations of 20 uM and 2uM, respectively. The cells were treated with Erastin or RSL3 for 24 h.

Once the cells attain a confluency level of 60–70%, they become suitable for transfection.During shRNA-HSF1 transfection, both shRNA-HSF1 and Negative RNA (Negative Control) were retrieved from refrigeration and subjected to centrifugation at a rate of 4000 rpm for a duration of 5 min.The transfection process involved the utilization of Lipofectamine 3000 reagent. Opti-MEM medium was used to dilute both Lipofectamine 3000 (Invitrogen, Carlsbad, CA, USA) and shRNA-HSF1 separately (2.5ul Lipo + 50ul Opti-MEM/ per well, 2.5ul shRNA + 50ul Opti-MEM/per well). Subsequently, the two reagents were combined in a 1:1 ratio and incubated at room temperature for 5 min. The follow-up experiments were conducted 48 h post-transfection. The HSPE1 plasmid was transfected using identical procedures.(HSF1 shRNA sequence: GCAGAAGCATGCCCAGCAA. HSPE1 plasmid sequence: F-AGCGAATTCGCCACCATGGCAGGACAAGCGTTT AG;R-TTTGTAGTCGGAT CCGTCTACGTACTTTCCAAGAA).

### DCFH-DA probe assay for ROS

For probe loading, DCFH-DA was added at a final concentration of 10 µM (1 mL per well), and the plate was incubated at 37 °C with 5% CO_2_ for 30 min and mixed every 3–5 min during this period. After 30 min, the cells were washed with serum-free medium thrice, and DCFH-DA that did not enter the cells was removed. A fluorescence microscope was used to photograph the cells. The cells were also digested with trypsin, neutralized in 3% FBS medium and collected in EP tubes in a total volume of 1 ml. Cells were detected by flow cytometry.

### MDA detection

The MDA levels in PC3 or LNCaP cells were evaluated through the utilization of an MDA colorimetric assay kit (Beyotime, S0131S, Shanghai) in accordance with the manufacturer's protocol.

### Western blot

After quantification by a BCA protein assay kit, the lysate (5 μl) was loaded onto a 10% SDS‒PAGE gel. Then, proteins were transferred to PVDF membranes using the wet transfer method after electrophoresis (Millipore, USA). The membranes were sealed with 5% skim milk at room temperature for 90 min, incubated with a primary antibody (HSF1, 1:1000, cell signaling, #4356) (rabbit anti-Hsp10 mAb, 1:10,000, abcam, ab108600) at 4 °C overnight, and subsequently incubated with anti-rabbit secondary antibody (GAPDH, 1:2000, Beyotime, A0208) at room temperature for 1 h.

### Cell viability assay

PC3 or LNCaP cells were subjected to treatment in 24-well plates, followed by trypsinization and staining with 0.5% Trypan blue for a duration of 3 min. The enumeration of viable cells was performed using a hemocytometer under a microscope. The concentration of XJB-5–131 (HY-129460, MCE, USA) added was 10 μM.

### Correlation analysis of HSF1 and pathways

The TCGA dataset was used to download RNA-sequencing expression profiles for PRAD, as well as clinical information (https://portal.gdc.com). R software GSVA package was used. The correlation between the HSF1 gene and pathway scores was analyzed by Spearman correlation. All the analysis methods and R packages were implemented using R version 4.1.0.0, and a p value < 0.05 was considered statistically significant.

### CHIP-qPCR assays

CHIP analysis was performed according to the CHIP Assay Kit's protocol (Beyotime Biotech, Shanghai, China). After the DNA was precipitated by HSF1 and control antibodies, PCR was performed to detect the HSPE1 promoter (Region1-F GACTCGGAGGCGGAAGAAAAA, Region1-R CTCAAGGGTCAAATCGCGTC; Region2-F2AACTACCCCTTCGTCCCCTC,Region2-R2 CGGCCGGCTTAGTCTA GTTC).

### Mitochondrial membrane potential assay

JC-1 staining kit(Beyotime Biotech, Shanghai, China) was used for mitochondrial membrane potential test. After staining with JC-1, the PC3 cells were analyzed by flow cytometry.

### In vivo experiment

Twenty 6-week-old BALB/c nude mice were divided into four groups: the NC group (n = 5), HSF1-shRNA group (n = 5), NC + RSL3 group (n = 5), and HSF1-shRNA + RSL3 treatment group (n = 5). The RSL3(dissolved in 50% PEG300 and 50% saline) treatment groups were subcutaneously injected with RSL3 at a dose of 100 mg/kg once weekly for two weeks. The mice in control group received an equivalent volume of the solvent.The tumor cell concentration was adjusted to 2 × 10^7^ cells/ml, and the inoculation volume was 100 µl. The cell suspension (without matrigel) was fully dispersed with a pipette, and 100 µl of the suspension was drawn with a 1-ml syringe and injected into the right axilla of mice. After approximately 2 weeks, the tumor size was recorded with a Vernier caliper, and tumor size was recorded twice a week. After 4 weeks, the tumor mass was removed, and its size and weight were recorded.

### Statistical analysis

Differences between the two groups were analyzed by Student’s *t* test, and P < 0.05 indicated statistical significance. All data analyses were performed using GraphPad Prism 9.0 software.

## Results

### Protein expression and functional enrichment analysis of high-risk prostate cancer

**Fig. 1:**
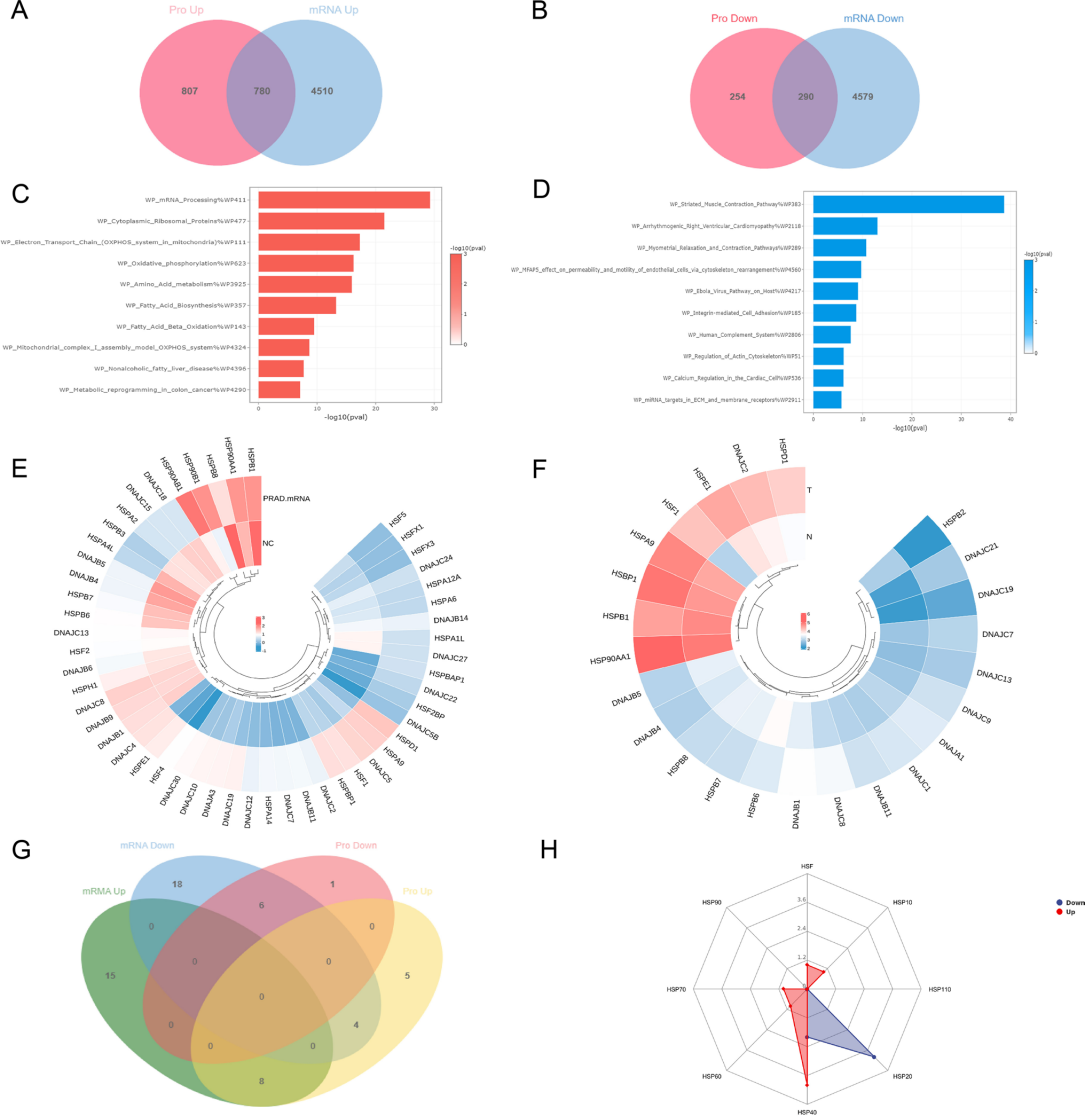
Proteomic analysis of high-risk prostate cancer. **A**, **B** Volcano map and heat map of differentially expressed protein. **C**, **D** Enrichment analysis of up-regulated and down-regulated protein WIKI pathways. **E** The heatmaps depicting the differential expression of Heat Shock Proteins (HSPs) at the mRNA level were obtained from the TCGA-PRAD database. **F** The heatmaps depicting the differential expression of Heat Shock Proteins (HSPs) at the protein level were obtained from our proteomic data. **G** The Venn graph shows intersection of differentially expressed HSPs in mRNA level and protein level. **H** Distribution of differentially expressed HSPs (According to molecular mass)

Proteomic analysis of 13 pairs of high-risk PC and normal adjacent tissues revealed that 1587 proteins were upregulated and 544 proteins were downregulated. Subsequently, the TCGA-PRAD database was utilized to examine the mRNA differences between high-risk PC and adjacent normal tissues. As shown in the Venn diagram, a total of 780 genes were coupregulated and 290 genes were codownre- gulated (Fig. 1A, B).

Pathway analysis of the differentially expressed genes (DEGs) showed that the coupregulated genes were significantly enriched in metabolic and oxidative phosphorylation-related pathways. The following Wiki pathways were significantly activated: Amino_Acid_metabolism, Fatty_Acid_Biosynthesis, Fatty_Acid_Beta_ Oxidation,Oxi dative_Phosphorylation, Electron_Transport_Chain (OXPHOS_system _in_mitochondria) and Mitochondrial_complex_I_assembly_model_OXPHOS_ systems pathways. In summary, Wiki pathway enrichment analysis revealed that metabolic reprogramming and the improvement of mitochondrial respiratory chain activity seemed to be important features in high-risk PC tissues compared to normal tissues (Fig. 1C). Conversely, the pathway of striated muscle contraction exhibited the highest degree of enrichment among the down-regulated Wiki pathways (Fig. 1D). Furthermore, the expression of heat-shock proteins (HSPs) was also displayed at both the mRNA and protein levels (Fig. 1E, F). The intersection of differentially expressed HSPs revealed 8 significantly upregulated HSPs (HSF1, HSPE1, DNAJC2, HSPD1, HSPA9, DNAJC7, DNAJB11 and DNAJC19) and 6 significantly downregulated HSPs (HSPB1, HSPB7, HSPB8, HSPB6, DNAJB4 and DNAJB5) (Fig. 1G). In order of increasing molecular mass, the upregulated HSPs included heat shock transcription factor-HSF1, HSP 10, HSP 40, HSP 60, and HSP 70. HSP20 and HSP40 were downregulated HSPs (Fig. 1H).

### Analysis of HSF1 expression in prostate cancer

**Fig. 2:**
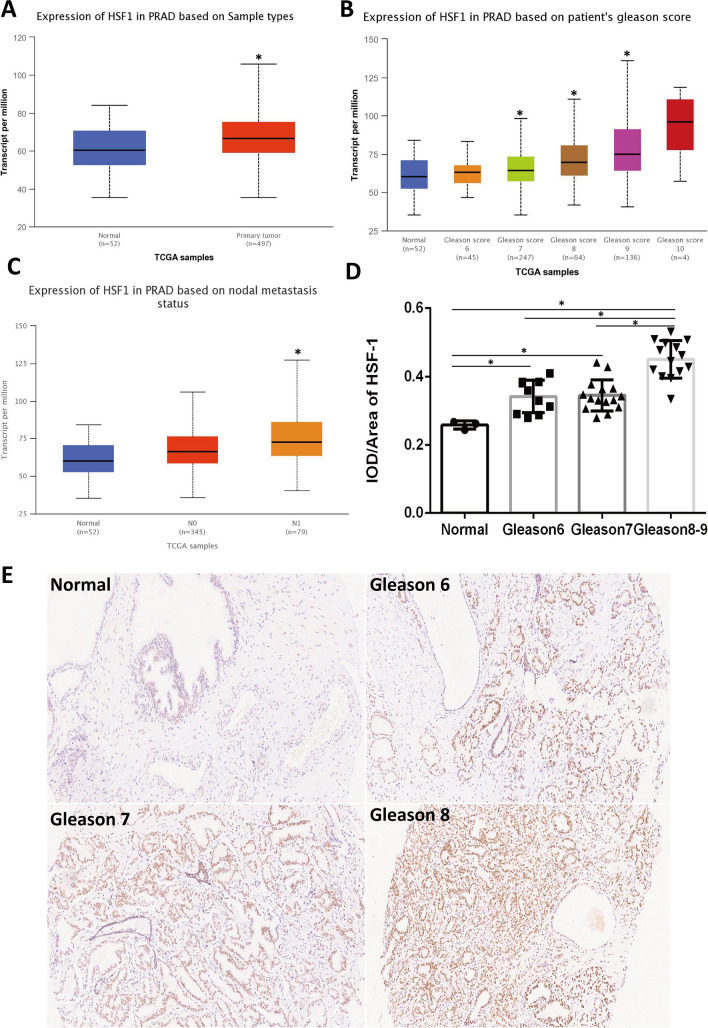
The expression of HSF1 in prostate cancer. **A**–**C** mRNA expression of HSF1 in TCGA (*P < 0.05). **D** Immunohistochemical semi-quantitative analysis (*P < 0.05). **E** Representative immunohistochemistry images of HSF1 in different prostate cancer tissues

TCGA database was further used to verify the mRNA expression levels of HSF1 in prostate cancer using the UALCAN platform [18]. The findings revealed a substantial increase in HSF1 mRNA levels in PC, which was positively correlated with Gleason score levels (Fig. 2A, B). Furthermore, HSF1 mRNA expression levels were significantly associated with metastasis (Fig. 2C).Subsequently, the expression levels of HSF1 protein were assessed in clinical tissues through the utilization of immunohistochemistry. The protein expression trend was consistent with that of mRNA, and semiquantitative analysis showed that HSF1 expression in subjects with high-risk PC(Gleason score ≥ 8) was significantly higher than that noted in subjects with medium-risk (Gleason score = 7) and low-risk PC (Gleason score = 6) (Fig. 2D). Representative immunohistochemical results are presented in Fig. 2E.

### Analysis of the correlation between HSF1 expression and clinical outcomes in prostate cancer

**Fig. 3:**
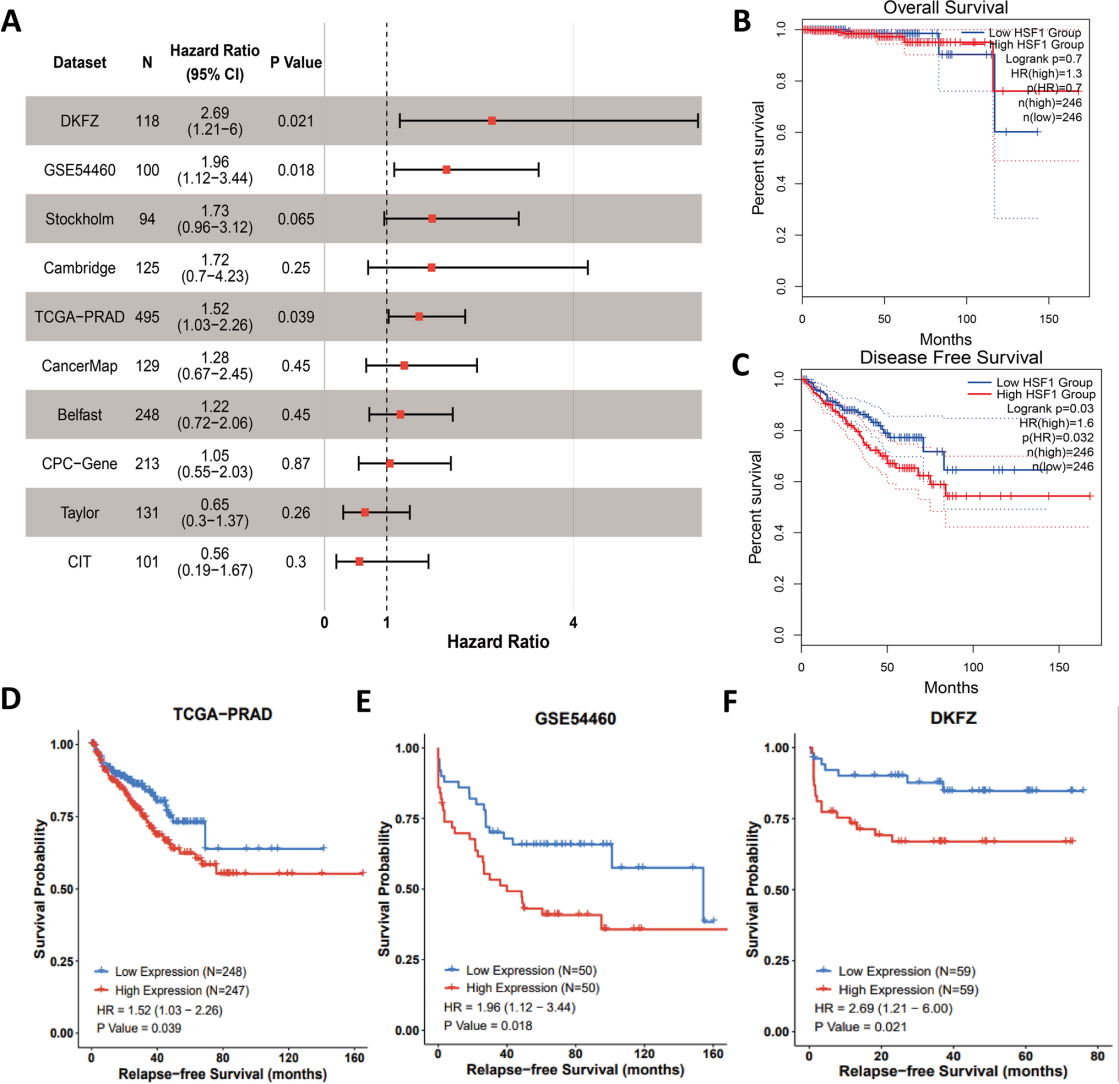
Survival analysis of HSF1. **A** Forest plot of the univariate Cox regression analysis in ten sets. **B**, **C** Kaplan–Meier survival curves in TCGA (OS, overall survival; DFS, disease free survival). **D**–**F** Kaplan–Meier survival curves in TCGA, GSE54460, and DKFZ (RFS, relapse free survival)

GEPIA (http://gepia.cancer-pku.cn/) database platform [19] was used to analyze the correlation between HSF1 gene expression and prognosis(overall survival and disease free survival), and the results showed no difference in overall survival but a significant difference in disease-free survival among prostate cancer patients with high HSF1 expression (Fig. 3B, C). In addition, the correlation between relapse free survival and HSF1 expression in 10 datasets was analyzed by PcaDB [20], the results showed that patients with high HSF1 expression exhibits significant differences in relapse-free survival among the TCGA, DKFZ and GSE54460 databases (Fig. 3A, D–F).

### SsGSEA of HSF1 in TCGA PRAD patients

**Fig. 4:**
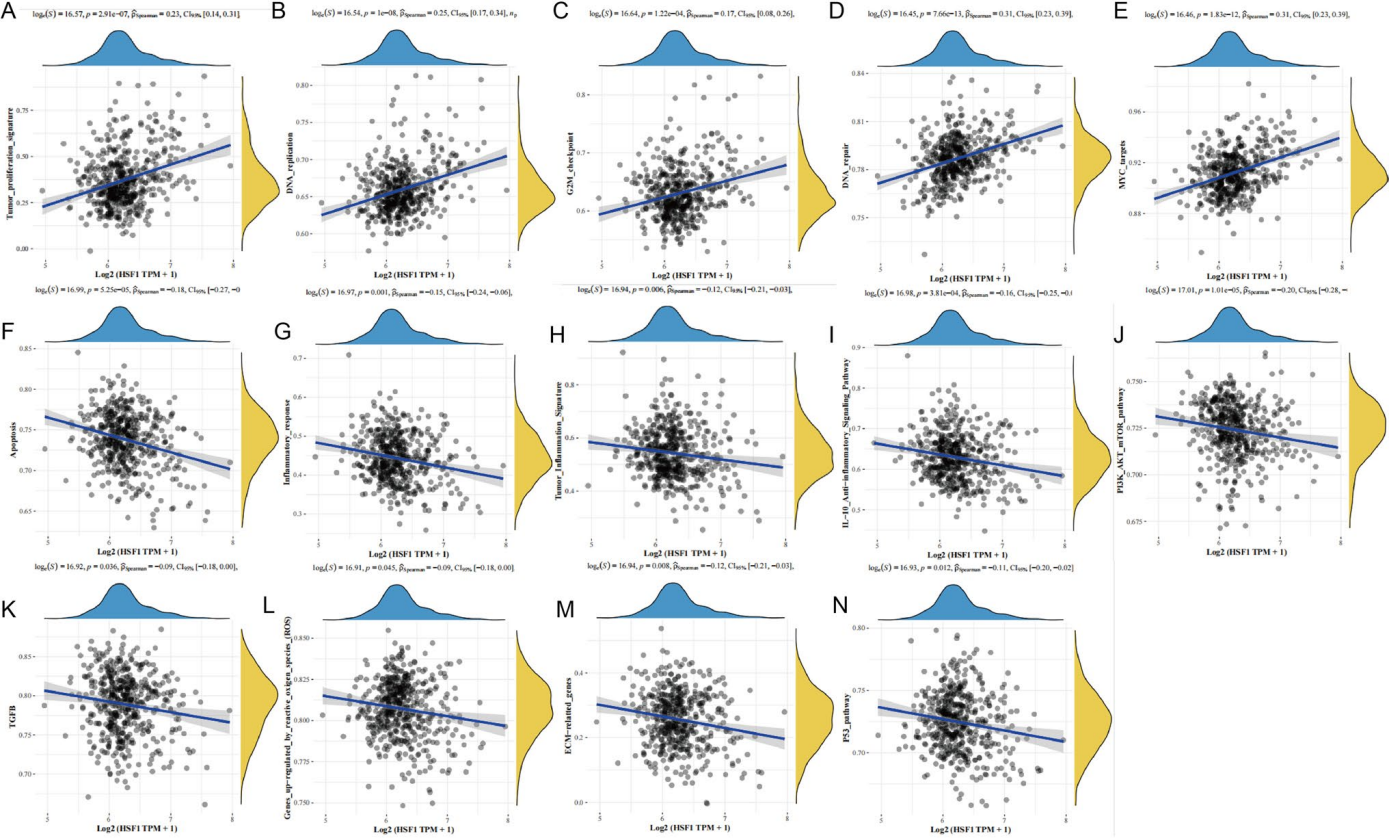
ssGSEA analysis of HSF1 and pathways, and.the correlations between individual gene and pathway score was analysed with Spearman. **A**–**E** The pathways that exhibit a positive correlation with HSF1 are presented. **F**–**N** The pathways that exhibit a negative correlation with HSF1 are presented

According to ssGSEA and pathway gene sets [21], the enrichment scores of relevant gene sets in each sample were calculated in turn. In order to determine the relationship between gene expression and pathway score, the correlation between gene expression and pathway score was calculated. Figure 4A–E shows pathways that were significantly positively associated with HSF1, including tumor_proliferation, DNA_ replication, G2M_checkpoint, DNA_repair, and MYC_targets. Figure 4F–N shows that pathways significantly negatively correlated with HSF1, including apoptosis, inflammatory response, PI3K-Akt-MTOR pathway, TGF-beta pathway, ECM-related genes, and P53 pathway. Thus, HSF1 is potentially mainly involved in the proliferation and anti-apoptosis processes of prostate cancer.

### Mutation and downstream pathway of HSF1

**Fig. 5:**
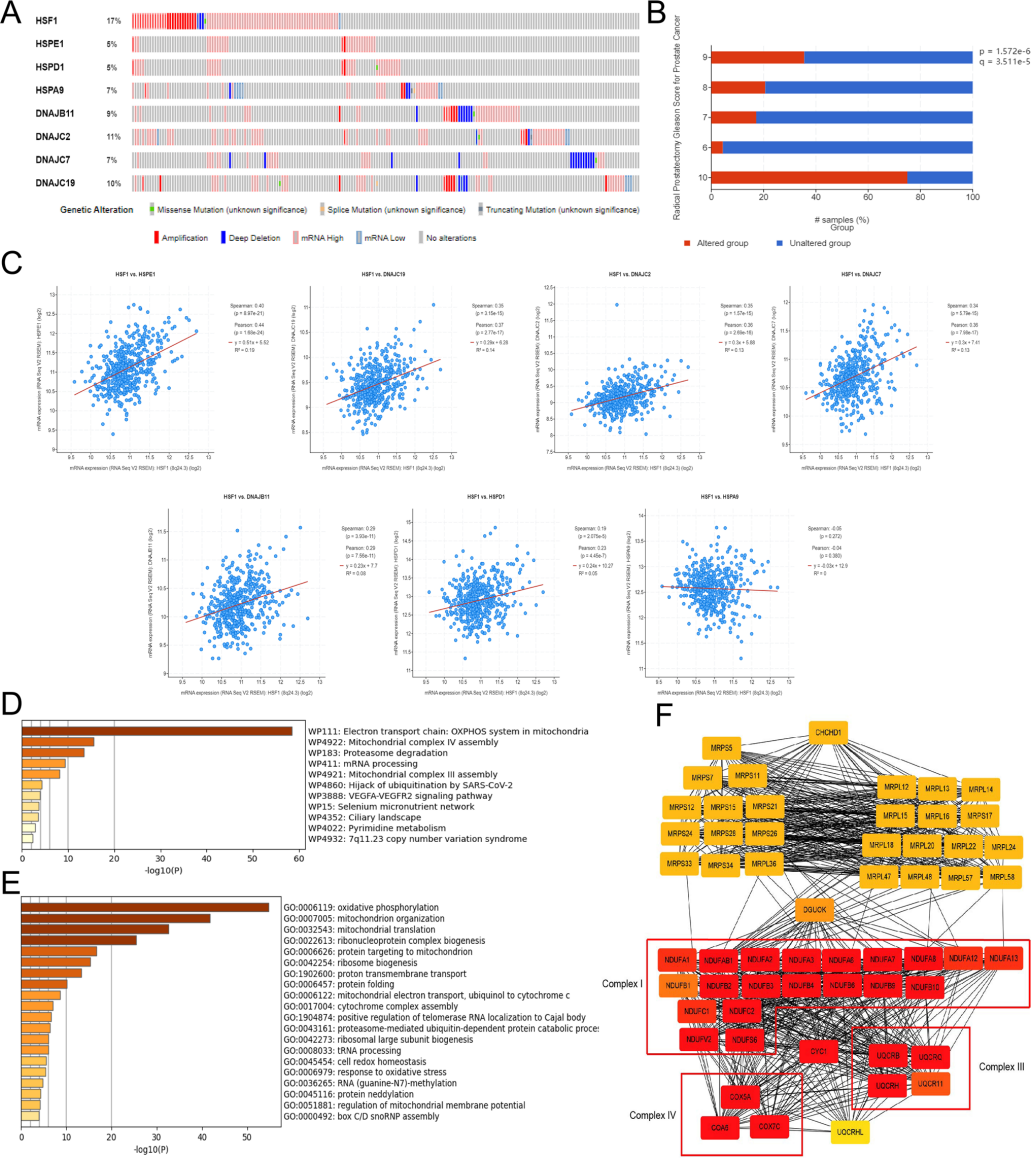
Mutation and downstream pathway of HSF1. **A** Mutation analysis in Cbioportal database. **B** Correlation between HSF1 mutation proportion and Gleason Score. **C** Correlation between HSF1 and HSPs expression levels. **D**, **E** Enrichment analysis of the top 300 HSPE1-positively correlated genes. **F** PPI analysis of HSPE1-positively correlated genes

We next analyzed the mutations of upregulated HSPs using the Cbioportal database (https://www.cbioportal.org/). The following mutational frequencies were observed: 17% for HSF1, 5% for HSPE1, 5% for HSPD1, 7% for HSPA9, 9% for DNAJB11, 11% for DNAJC2, 7% for DNAJC7 and 10% for DNAJC19 (Fig. 5A). Among them, the gene with the highest mutation rate was HSF1, accounting for 17% in PC, and the primary type of mutation was amplification. The correlation between HSF1 mutation status and clinical Gleason score was also analyzed (Fig. 5B). The higher the Gleason score, the higher the proportion of patients with HSF1 mutations. This finding was statistically significant (p < 0.001). This finding suggested that HSF1 mutation was closely associated with PC progression. In addition, we searched for the possible downstream effector molecules by which HSF1 regulates ferroptosis. Given that HSP expression is often regulated by HSF1, we first analyzed the correlation between HSF1 and HSPE1, HSPD1, HSPA9, DNAJB11, DNAJC2, DNAJC7 and DNAJC19 in TCGA-PRAD cohort using the cBioPortal database (Fig. 5C). The results indicated that HSF1 expression was significantly correlated with HSPE1, HSPD1, DNAJB11, DNAJC2, DNAJC7 and DNAJC19. Among them, the correlation between HSF1 with HSPE1 was the highest (Pearson = 0.44, P = 1.68e−24). To further explore the functions of HSPE1 in PC, enrichment analysis of the top 300 HSPE1-positively correlated genes was performed. Enrichment analysis of the WIKI and GO pathways showed that HSPE1 mainly participates in the electron transport chain:OXPHOS system in mitochondria and the assembly of the mitochondrial complex (Fig. 5D,E). These results were similar to those obtained for DEG analyses as presented in Fig. 1C. Protein–protein interaction analysis (PPI) analysis showed that HSPE1 is coexpressed with genes belonging to OXPHOS complexes I/III/IV (Fig. 5F). These results suggest that HSF1 may affect the mitochondrial oxidative phosphorylation system by targeting OXPHOS through HSPE1 and that the HSF1-HSPE1-OXPHOS network potentially represents an important regulatory mechanism in high-risk PC.

### HSF1 can directly regulate HSPE1 expression in prostate cancer

**Fig. 6:**
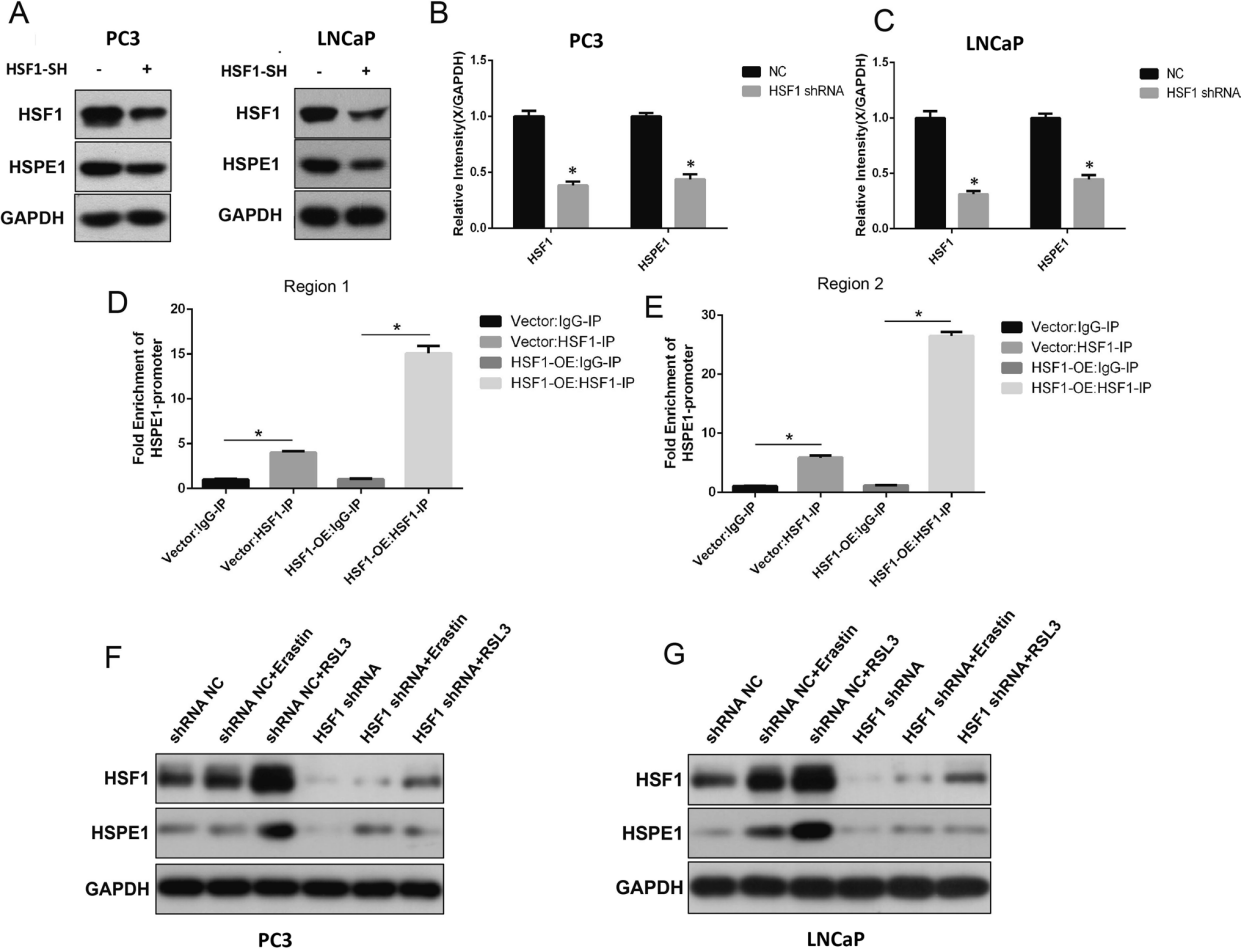
HSF1 can directly regulate HSPE1 expression in prostate cancer. **A**–**C** Expression levels of HSF1 and HSPE1 after HSF1 shRNA knockdown were tested by WB (*P < 0.05). **D**, **E** ChIP-qPCR assay of HSF1 binding to the HSPE1 promoter (*P < 0.05). **F**, **G** Expression levels of HSF1 and HSPE1 after treated with Erastin or RSL3 were tested by WB

The prostate cancer cell lines PC3 and LNCaP were used in vitro. Western blot results showed that after HSF1 knockout, HSF1 protein expression level decreased significantly and was accompanied by a decrease in HSPE1 expression (Fig. 6A, C).Then, ChIP-qPCR was performed(Fig. 6D, E). Anti-IP-HSF1 pull-down was significantly enriched with promoter fragments (Region 1 and Region 2) of HSPE1 in PC3 cell line. This result is shown more dramatically in cells that overexpress HSF1(HSF1-OE group). All these data implied that HSF1 can directly bind to promoter of HSPE1 and regulate its expression. In addition, we checked the expression level of HSF1 and HSPE1 after Erastin or RSL3 treatment both in PC3 and LNCaP cell lines. Upon treatment with ferroptosis-inducers, the expression levels of HSF1 and HSPE1 were significantly increased,especially in the RSL3 treatment group(Fig. 6F,G).This result might be a protective mechanism, which can protect the PC against ferroptosis.Thus, knockdown of HSF1 may be an important way to increase the sensitivity to ferroptosis in PC.

### HSF1 affects oxidative stress and lipid peroxidation levels by regulating HSPE1 in vitro

**Fig. 7:**
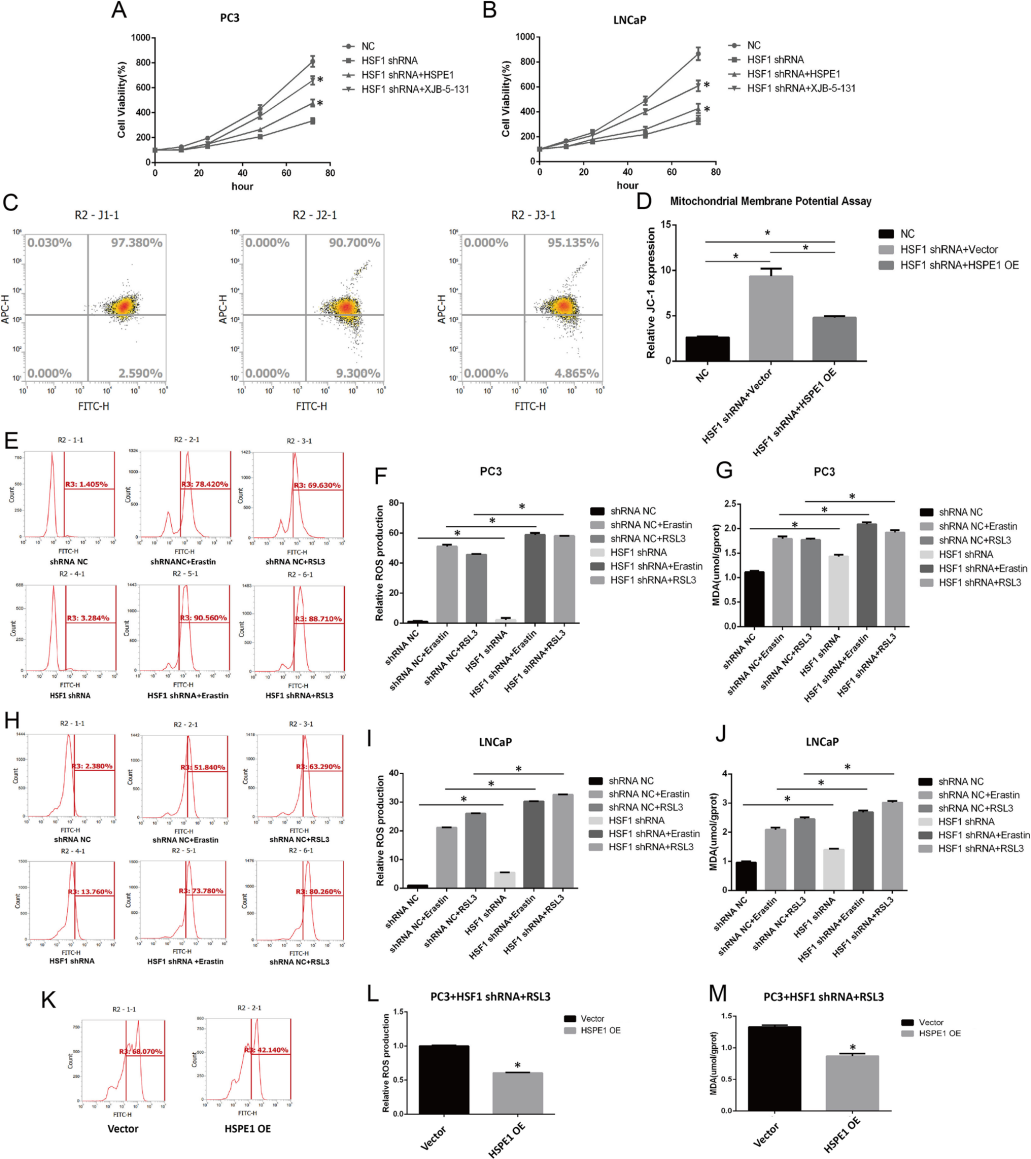
HSF1 affects oxidative stress and lipid peroxidation levels by regulating HSPE1 in vitro. **A**, **B** CKK-8 assay after HSF1 knockout (*P < 0.05). **C**, **D** Mitochondrial membrane potential assay in PC3 cell line via JC-1 staining and flow cytometry (*P < 0.05). **E**–**G** ROS and MDA assays in PC3 cell lines after HSF1 knockout (*P < 0.05). **H**–**J** ROS and MDA assays in LNCaP cell lines after HSF1 knockout. **K**, **M** ROS and MDA assays in PC3 cell lines after HSPE1 overexpression (*P < 0.05)

The CKK8 results showed that the cell activity of shRNA-HSF1 group was significantly decreased, but could be improved by ferroptosis inhibitor XJB-5–131 (a ferroptosis inhibitor targeting mitochondria ROS and electron scavenger), suggesting that shRNA-HSF1 could induce ferroptosis. Besides, overexpression of HSPE1 can also rescue the cell death caused by shRNA-HSF1 (Fig. 7A, B).Mitochondrial damage can lead to the decrease of the mitochondrial membrane potential (Δψm) and contribute to ROS accumulation and ferroptosis. In JC-1 assay, the increase of green fluorescence suggested the decrease of membrane potential. The results showed that the green fluorescence increased after HSF1 knockout in PC3 cell line (the proportion of cells in the fourth quadrant increased in flow cytometry), and the overexpression of HSPE1 could rescue the mitochondrial damage caused by HSF1 knockout (Fig. 7C, D). Besides, ROS and MDA are two important indicators of oxidative stress and ferroptosis. ROS affect lipids and lead to lipid peroxidation, consequently producing MDA, a crucial driving force of ferroptosis. We further compared ROS levels in shHSF1-transfected cell lines. ROS levels increased significantly in HSF1 knockout group and RSL3- or Erastin-treated groups than in paired control groups. Of noted, ROS levels were most significantly increased in the HSF1 shRNA + RSL3 or HSF1 shRNA + erastin combination treatment groups (Fig. 7E, F). The same trend was observed for MDA assay results (Fig. 7G). Moreover, similar results were observed in LNCaP cells (Fig. 7H, J). The restoration of HSPE1 levels in shHSF1-transfected cell lines treated with RSL3 resulted in noteworthy decreases in ROS and MDA levels during the rescue experiment (Fig. 7K–M). In conclusion, HSF1 may alleviate ROS and MDA levels to enhance the resistance of prostate cancer cells to ferroptosis by regulating HSPE1.

### HSF1 knockdown increases the therapeutic efficacy of prostate cancer cells to ferroptosis inducer in vivo

**Fig. 8:**
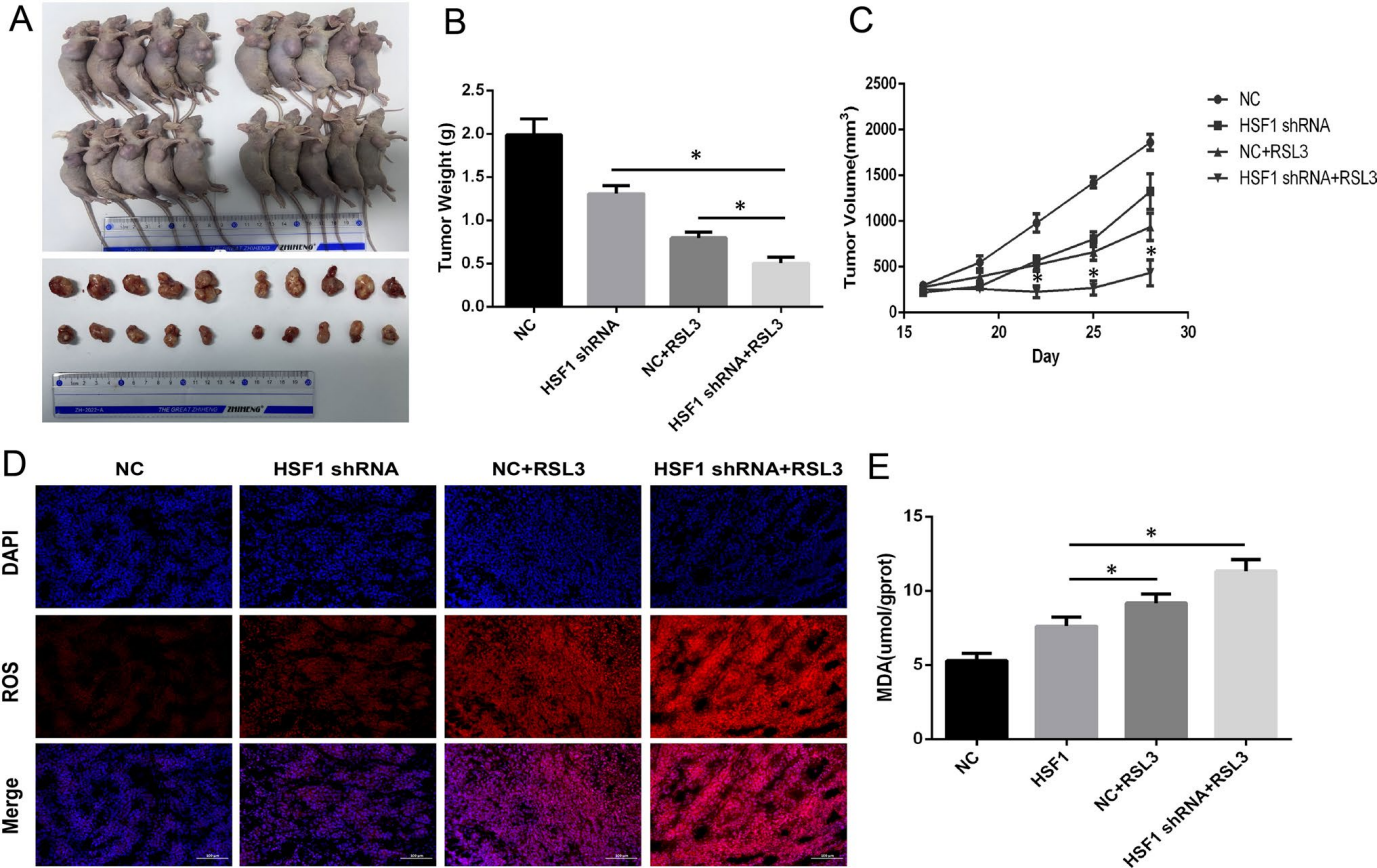
Experiments in vivo. **A**–**C** Tumor weight(g) and tumor growth curves (mm^3^) of mice subcutaneously inoculated with PC3 cells (* P < 0.05). **D**, **E** ROS staining and MDA assay in tumor tissues were performed after 4 weeks (*P < 0.05)

RSL3 is a potent inducer of ferroptosis. Then, we assessed therapeutic efficacy of RSL3 after HSF1 knockout in vivo. The results showed that HSF1 knockout alone significantly reduced the tumor volume compared to that in the control group, and the same results were observed in the RSL3 treatment and HSF1 shRNA + RSL3 treatment groups. There was a remarkable difference in efficacy between the combination group and the control group regarding tumor volume suppression (Fig. 8A, C).To further probe the mechanism of the synergistic effect, we next compared the characteristics of ferroptosis in tumor tissue between different groups. The results showed that ROS and MDA levels were significantly increased in tumor tissue after HSF1 knockout, and the highest ROS and MDA levels were noted in the HSF1 shRNA + RSL3 treatment group (Fig. 8D, E). These results suggest that HSF1 knockout potentially promotes the therapeutic efficacy of prostate cancer to RSL3 treatment by increasing ferroptosis.

## Conclusion

PC is the second most common malignancy and topped the list of newly diagnosed malignancies among men in the United States in 2020 [22]. About 15% of patients diagnosed with PC are considered high risk with a high mortality rate. It has become clear that determining risk solely based on the T, N, and M classification, which does not include the Gleason score or PSA, leads to many limitations. D 'Amico et al. first proposed a more reliable “high risk” model, which was defined as a PSA ≥ 20 ng/mL or a Gleason score ≥ 8 or a clinical T stage ≥ cT2c,which has been adopted by the American Urological Association (AUA).As well as this, the Radiotherapy Therapy Oncology Group (RTOG) also developed a classification to correlate specific baseline factors with overall survival and cause-specific survival, including (1) Gleason score ≥ 8 or (2) Gleason score = 7 plus clinical T stage ≥ cT3 or positive lymph node [23]. To further refine risk, other specific biologic determinants, such as Ki-67, the PI3K/PTEN signaling axis, and the alteration of DNA copy numbers, were measured in diagnostic tumor biopsy samples in addition to the Gleason score alone [24, 25]. In our study, a comparison between patients with Gleason scores ≥ 8 and those with Gleason scores ≤ 6 or 7 revealed significant increases in HSF1 mRNA and protein expression. This result suggests that HSF1 expression in PC is significantly positively correlated with the Gleason score and thus exhibits potential as a new risk classification index.

Metabolic reprogramming is an important feature of tumors, and lipid metabolism is closely related to the progression and metastasis of prostate cancer. Studies have shown that increased fatty acid uptake and de novo synthesis are associated with the tumor energy supply in PC [26]. In addition, obesity and metabolic syndrome are associated with the poor survival of PC patients [27]. In this study, WIKI pathway analysis showed a significant enrichment in mitochondrial electron transport, oxidative phosphorylation, fatty/amino acid metabolism in high-risk PC. These results suggest that altered cell energy metabolism is a crucial hallmark of PC. It is worth noting that the electron transport chain is not only the basis of energy generation but also an important source of ROS. As high ROS levels are generally detrimental to cells, PC cells potentially possess complicated antioxidant mechanisms to protect against oxidative stress.

As a molecular chaperone, HSP participates in the folding of proteins involved in normal metabolic processes. The ability of cells to respond to stress by increasing HSP levels depends on the activity of HSF1, and HSF1 promotes the transcription of HSPs by binding to their promoters [28, 29]. Accumulating evidence clearly indicates that high HSF1 levels effectively maintain protein stability in the hypermetabolic environment of tumor cells, and cancer cells have also been described as having a "nononcogenic" dependence on HSF1. In this study, we found that HSF1 was significantly upregulated in high-risk PC. After screening the downstream proteins of HSF1, we found that HSPE1 exhibited the strongest correlation with HSF1 in prostate cancer, and HSPE1 mainly functions in maintaining the mitochondrial ETC. Increasing evidence suggest that ferroptosis is a type of mitochondria-related cell death that is driven by increased ROS levels in the body, and mitochondrial damage is often associated with ROS stress and cell death [30, 31]. Studies have also shown that obstructions in respiratory chain electron flow result in electron leakage and reactive oxygen species (ROS) formation [32, 33]. Given that bioinformatics analysis has shown that HSPE1 is mainly involved in the function of the ETC in mitochondria, HSF1/HSPE1 potentially represent crucial molecules affecting ROS levels in prostate cancer. Our further experiments showed that HSF1 knockdown downregulates HSPE1 and increases ROS and MDA levels in PC3 cells, resulting in increased sensitivity to ferroptosis induced by erastin or RSL3, and the effect could be rescued by HSPE1 overexpression. These results indicated that HSF1 may maintain the metabolism and ROS stability of PC via the upregulation of HSPE1.

The mechanism by which HSF1 affects ferroptosis via HSPE1 in PC has not been reported. In 2012, Dixon first reported that erastin induced fibrosarcoma tumor cell death characterized by mitochondrial constriction and an increased bilayer membrane density [34]. Moreover, this pattern of cell death is characterized by iron-dependent lipid peroxidation, which is regulated by the cystine transport pathway [35]. This death mechanism is also named ferroptosis and is caused by the adherent accumulation of lipid peroxides and lipid reactive oxygen species. Studies have shown that cell sensitivity to ferroptosis is related to a variety of biological pathways, including amino acid and glutathione metabolism, iron metabolism and fatty acid metabolism [36]. Considering the high level of activation of metabolism-related pathways in PC, ferroptosis might play an important role in progression. This study employed RSL3 and Erastin as ferroptosis inducers in PC. These two agents are recognized as classic inducers of ferroptosis. Specifically, RSL3 functions as an inhibitor of glutathione peroxidase 4 (GPX4), thereby reducing the expression of GPX4 protein and inducing ferroptosis.Besides, Erastin acts by inhibiting voltage-dependent anion channels (VDAC2/VDAC3) and promoting oxidation, which results in the accumulation of endogenous reactive oxygen species [37, 38].

At present, androgen deprivation therapy (ADT) is an important treatment in PC, but the patients gradually becomes less sensitive to this treatment and ultimately converts to castration-resistant prostate cancer (CRPC). Studies have shown that ferroptosis inducers might represent a novel therapeutic approach for PC [39, 40]. As a result, the identification of molecular mechanisms that increase susceptibility to ferroptosis will contribute to targeted prostate cancer therapies. In this study, to confirm the effect of the combination, an in vivo assay was also performed. The results showed that cotreatment with shRNA-HSF1 and RSL3 significantly reduced tumor size compared with the effects of shRNA-HSF1 or RSL3 alone. This finding indicates that HSF1 intervention may represent an effective method to improve the efficacy of ferroptosis-related targeted therapy.

In conclusion, the results of this study improve our understanding of the relationship between HSF1 and PC. On the one hand, we analyzed HSF1 mRNA and protein expression in PC and found that its expression levels potentially serve as a supplementary indicator of risk. On the other hand, HSF1 may alleviate ROS and MDA levels to enhance the resistance of PC cells to ferroptosis by regulating HSPE1. In summary, our results suggest that HSF1 can serve as a risk-grade indicator and prognostic biomarker for PC and that combination therapy consisting of targeting HSF1 and ferroptosis inducers might represent a novel therapeutic approach for PC in future.

## Data Availability

If reasonably requested, the corresponding author will provide the data sets that were used and/or analyzed in this study.
